# *In vitro* and *ex vivo* approach for anti-urolithiatic potential of bioactive fractions of gokhru with simultaneous HPLC analysis of six major metabolites and their exploration in rat plasma

**DOI:** 10.1080/13880209.2016.1266671

**Published:** 2016-12-16

**Authors:** Ikshit Sharma, Washim Khan, Sayeed Ahmad

**Affiliations:** Department of Pharmacognosy and Phytochemistry, Faculty of Pharmacy, Bioactive Natural Product Laboratory, Jamia Hamdard (Hamdard University), New Delhi, India

**Keywords:** Tribulus terrestris, fractionations, aggregation, turbidity, nucleation, HPLC quantification

## Abstract

**Context:** *Tribulus terrestris* L. (Zygophyllaceae) fruits have long been used in traditional systems of medicine for the treatment of various urinary diseases including urolithiasis.

**Objective**: To explore the anti-urolithiatic potential of gokhru and to develop an analytical method for quantitative estimation of metabolites for its quality control.

**Materials and methods:** Aqueous extract of gokhru fruit was prepared through maceration followed by decoction to produce a mother extract, which was further used for polarity-based fractionations. *In vitro* and *ex vivo* anti-urolithiatic activity of mother extract and fractions at different concentration (100–1000 μg/mL) were carried out using aggregation assay in synthetic urine and in rat plasma, however, nucleation assay for 30 min was done using confocal microscopy. A simultaneous HPLC method has been developed for quantification of diosgenin, catechin, rutin, gallic acid, tannic acid and quercetin in mother extract and in fractions.

**Results:** The extraction resulted in 14.5% of w/w mother extract, however, polarity-based fractionation yielded 2.1, 2.6, 1.5, 1.3 and 6.1% w/w of hexane, toluene, dichloromethane (DCM), *n*-butanol and water fractions, respectively. *In vitro* and *ex vivo* studies showed a significant anti-urolithiatic potential of *n*-butanol fraction. Further, HPLC analysis revealed significantly (*p* < 0.01) higher content of quercetin (1.95 ± 0.41% w/w), diosgenin (12.75 ± 0.18% w/w) and tannic acid (9.81 ± 0.47% w/w) in *n*-butanol fraction as compared to others fractions.

**Discussion and conclusion:** *In vitro* and *ex vivo* studies demonstrated potent anti-urolithiatic activity of *n*-butanol fraction which can be developed as new phytopharmaceuticals for urolithiasis. HPLC method can be used for quality control and pharmacokinetic studies of gokhru.

## Introduction

*Tribulus terrestris* L. (Zygophyllaceae) fruits, a natural herb also known as gokhru, have long been used in both Indian and Chinese systems of medicine for the management of the various diseases. It has been reported to have diuretic (Al-Ali et al. 2003), aphrodisiac (Adaikan et al. 2001), immunomodulatory (Tilwari et al. 2011), antilithiatic (Aggarwal et al. 2012; Saxena & Argal 2015), antidiabetic (Li et al. 2002), hypolipidaemic, antidepressant (Deole et al. 2011), cardiotonic, anti-inflammatory, hepatoprotective, anticancer, antibacterial, anthelmintic and antispasmodic activities (Chhatre et al. 2014). In India, fruits are commonly used in folklore to treat urolithiasis. So far, its diuretic properties have been documented in the literature and is actively used in various drug formulations for kidney stone (Al-Ali et al. 2003; Dixit et al. 2014). The anti-urolithiatic activity of gokhru was suggested due to the presence of quercetin and kaempferol in chloroform fractions of methanolic extracts, which attributed glycolate oxidase (GOX) inhibition resulting in oxalate synthesis inhibition (Shirfule et al. 2011). Bearing in mind the potential of gokhru extracts to treat kidney, the present study was proposed to find out the major metabolites of gokhru responsible for its pharmacological activity by testing its polarity-based fractions against aggregation and nucleation assay followed by analysis of metabolites. In addition to kaemferol, other constituents found in gokhru are diosgenin, neogitogenin, hecogenin, kaemferol-3-glucoside, quercetin 3-*O*-rutinoside (Matin et al. 2008), tribuloside and caffeoyl (Chhatre et al. 2014).

Analysis of metabolites through a sensitive analytical method has now become one of the desired trends for quality control, pharmacokinetics and metabolomic analysis to find the mechanistic approach of bioactivity. However, very few HPLC analyses of gokhru have been documented for the quantitative determination of flavonoids (Kumar 2012; Shiquan & Ruihai 2015) and diosgenin (Gupta et al. 2012; Yu 2008). To our best knowledge, there are no simultaneous HPLC analytical methods for the quantitative estimation of flavonoids, phenolics, polyphenolics and saponins available till available. Keeping in mind the limitations of the reported methods and complexity of extracts/fractions, a novel HPLC method was proposed for simultaneous quantification of six common metabolites of gokhru in bioactive fractions. Newly developed HPLC method can perform both qualitative and quantitative analysis of these metabolites simultaneously. The method was also applied for the determination of recovery of targeted metabolites in a biological matrix so that the same method can be explored for pharmacokinetics of gokhru.

## Materials and experimental methods

### Chemicals

Catechin (≥99.0%), diosgenin (≥93.0%), gallic acid (≥97.5%), quercetin (≥95%), rutin (≥94%) and tannic acid (≥98.0%), were procured from the local dealer of Sigma-Aldrich, New Delhi, India. Analytical grade and HPLC grade solvents were procured from Merck Life Science Private Limited, Bengaluru, India. All the solutions for analysis were freshly prepared.

### Preparation of plant extract and its bioactivity-guided fractionations

Dried and powdered fruits of gokhru (500 g) were extracted by boiling with demineralized water (1:16, w/v) until the volume was reduced to one-eighth of the original volume. The extract was filtered and the filtrate was evaporated on a boiling water bath until constant weight was obtained to give the decoction extract. The aqueous (mother) extract thus obtained was suspended in double-distilled water (1.0 g/10 mL) and sonicated for 15 min at 45 °C. Polarity-based fractionation of prepared aqueous suspension was done using equal proportions of hexane, toluene, dichloromethane (DCM) and *n*-butanol (three times each). The aqueous suspension left after fractionation was evaporated to dryness. The extractive values and % yields of different fractions were calculated and stored at 4 °C for bioactivity and quality control analysis.

### Anti-urolithiatic activity of Gokhru extracts and its fractions

#### *In vitro* aggregation assay

Inhibition of aggregation of oxalate crystallization assay by using plant extract was carried out according to the protocol described by Atmani and Khan (2000) with slight modification. This model includes the study of crystallization without inhibitor and with it, in order to assess the inhibiting capacity of the plant extracts/fractions used. Various concentrations of calcium chloride (CaCl_2_) and sodium oxalate (Na_2_C_2_O_4_) ranging from 1–40 mmol/L were examined in order to standardize their concentrations through crystallization assay. The final concentrations of calcium chloride and sodium oxalate were selected based on their sensitivity in spectrophotometer and turbidity of the solution. A solution of calcium chloride and sodium oxalate were prepared at the final concentrations of 6.0 and 6.5 mmol/L, respectively, in a buffer containing Tris 0.05 mol/L and NaCl 0.15 mol/L at pH 6.5. The calcium chloride solution (950 μL) mixed with 100 μL of herb extracts at the different concentrations (100–1000 μg/mL). Crystallization was started by adding 950 μL of sodium oxalate solution. In case of control experiment, 100 μL of buffer was added to calcium chloride solution. The temperature was maintained at 37 °C throughout 1 h incubation period. The optical density (OD) of the crystallized suspension was monitored at 620 nm. The percentage aggregation inhibition was then calculated by comparing the turbidity in the presence of extract with that obtained in the control using following formula (Masao et al. 2000).

$$\%\;\mathrm{inhibition}\;=\;({1-\mathrm{Turbidity}}_{\mathrm{sample}}{/\mathrm{Turbidity}}_{\mathrm{control}})\;\times \;100$$

The growth of crystals was expected due to the following reaction:

$${\mathrm{CaCl}}_{2}\;+\;{\mathrm{Na}}_{2}C_{2}O_{4}\to {\mathrm{CaC}}_{2}O_{4\;}+{}_{\;}2\mathrm{NaCl}$$

#### Ex vivo turbidity assay

The oxalate crystal inhibition potential of gokhru extract and fractions were also carried out in rat plasma to provide the biological environment. The plasma sample was diluted with equal volume of 12 mmol/L calcium chloride and sodium oxalate, separately. Resulting plasma solution containing either calcium chloride or sodium oxalate was used for nucleation assay. Plasma (950 μL) containing sodium oxalate (5.0 mmol/L) was mixed with gokhru extracts/fractions (100 μL) at different concentrations (100–1000 μg/mL). Crystallization was carried out by adding 950 μL of plasma containing calcium chloride (5.0 mmol/L). The mixture solution was incubated for 1 h and the temperature was maintained at 37 °C. The OD of the crystallized suspension was monitored at 620 nm. The inhibition potential of gokhru extracts was estimated by comparing with control. The percentage aggregation inhibition was then calculated by comparing the turbidity in the presence of the extract with that obtained in the control using the formula given above (Masao et al. 2000).

#### Nucleation assay in confocal microscope

All reaction solutions were pre-heated to 37 ± 0.2 °C. Glass-bottomed Petri dishes (polystyrene dish: diameter: 35 mm; glass bottom: grade no.: 1.5; diameter: 10 mm) pre-heated and assembled on the stage of a confocal microscope (Leica Microsystem, GmbH; Model-ERG0PLATTE DMI). Oxalate solutions added to the dishes followed by buffer (for control)/mother extract or *n*-butanol fraction (as a test sample) and calcium solutions. To obtain high contrast image, equimolar concentrations of calcium and oxalate of 1.0 mM were mixed. The total reaction volume of solution was 1.0 mL. Crystallization was allowed to proceed for 30 min. All precipitates were scanned and imaged using a helium/neon-laser (wavelength: *λ* = 632.8 nm), a 63× plane achromat oil immersion objective, a 90/10 mirror and the LSM AF Lite Software version 1.0.0 (Leica Microsystem, GmbH). Before every experiment, a pre-alignment of the microscope was carried out and the focus adjusted to the interface between glass and reaction solution. After adding the calcium chloride (Ca^2+^), nucleating and growing crystals were imaged every 20 s for 30 min. To maintain the focal plane at the crystal–glass interface, a fine tuning of the focus was conducted during imaging. Images captured using helium/neon-laser represents surfaces of crystals generated at the crystal glass interface. Shape and size of generated crystals was evaluated and for growth kinetics, three image sequences of replicate samples were taken by measuring through a ruler (Grohe et al. 2006).

### Development of HPLC method for simultaneous estimation of diosgenin, catechin, rutin, gallic acid, tannic acid and quercetin

#### Instrumentation

Alliance HPLC system (e2695 Separation module, Waters, New Delhi, India) with a gradient pump integrated with variable wavelength programable photodiode array detector was employed for the investigation and chromatographic separation was performed on Hupersil 5.0 μ C18 (ODS) column (150 × 4.6 mm, Phenomenex p, Hyderabad, India) whereas Mettler Toledo balance (Model: AB204-S) was used for all weighing.

#### Chromatographic conditions

Chromatographic separation was achieved on Hupersil 5.0 μ C18 (ODS) column using mobile phase composition of 0.5% v/v formic acid in water (A) and acetonitrile (B) at a flow rate of 1.0 mL/min in gradient elution program (Initially 100% A, 0–5 min: 90% A, 5–10 min: 70% A, 10–15 min: 30% A, 15–20 min: 2% A, 20–22 min: 100% A). The formic acid solution in water was filtered through 0.45 mm and degassed before use. Detection was carried out in a scanning mode with 3D channel by PDA detector and specific wavelengths were set at 254 nm (for rutin and quercetin), 278 nm (for gallic acid, tannic acid, and catechin) and 285 nm (for diosgenin). The identities of these compounds were established by comparing retention times of the sample solution with those of analytical grade standard solutions. The total run time was 25 min and the respective peak areas were applied to quantify the studied metabolites. The proposed developed method has been validated as per the ICH guideline, similar to methods reported by the laboratory (Kamal et al. 2012).

#### Preparation of standard stock and working solution

Accurately weighed quantity of all the standards to be analyzed (10 mg) was transferred to 10.0 mL volumetric flask. Then, small amount of methanol was added and ultrasonicated for 5 min and diluted upto the mark with methanol (concentration: 1.0 mg/mL of each standard). From the stock solution, 1.0 mL was pipetted out into 10 mL volumetric flask and made up the final volume with methanol (100 μg/mL). In the same way, for linearity assay, standard solution ranging from (0.5–100 μg/mL) were prepared for the above-mentioned solution using mobile phase as a diluent to increase the sensitivity of the analytical method.

#### Sample preparation for HPLC analysis of diosgenin, catechin, rutin, gallic acid, tannic acid and quercetin in blood

Blood was collected from rat and centrifuged for 10 min at 4700 *g* at 4 °C. The supernatant was transferred into reaction cups in aliquots and drug mixture solutions were spiked. The serum sample was spiked with drug mixture solution to get a concentration of 500 ng/mL. The simple conventional sample preparation method was applied for extraction of targeted markers through liquid–liquid extraction. To 500 μL of the spiked serum sample, 100 μL of methanol was added for protein precipitation. Precipitated protein was separated by centrifugation and supernatant was collected. To 100 μL of supernatant, 300 μL of organic solvents was used. Ethyl acetate, methyl-*tert*-butyl-ether, methanol and mixture of ethyl acetate with isopropyl alcohol (1:1, v/v) were employed to screen the best extracting solvents through liquid–liquid extraction. Another liquid mixture of ethyl acetate, methyl-*tert*-butyl-ether and acetonitrile with a ratio of 1:1:1, v/v/v was also used for liquid–liquid extraction. Further, protein-free serum samples were acidified or basified or both acidified and basified then extracted with best extracting solvents. Dilute hydrochloric acid (0.1 N) and sodium hydroxide (0.1 N) solutions in methanol (20 μL each for 100 μL serum) were used for acidification and basification of serum samples, respectively (Oh & Lee 2014). The organic layer was collected and evaporated to dryness under N_2_. The dried extract was re-constituted in 150 μL of HPLC grade methanol and filtered through 0.45 μM PTFE membrane filter. The samples were stored at −20 °C prior to analysis.

### Validation of developed HPLC method

The chromatographic method was validated for selectivity, linearity range, detection and quantification limits, accuracy and precision according to the ICH guidelines, similar to methods reported by the laboratory (Kamal et al. 2012).

#### System suitability

To evaluate system suitability of the method, retention time (*R*_t_), theoretical plates, tailing factor, height equivalent theoretical plate (HETP), capacity factor, peak asymmetry and retention times of six replicate injections of each standard of concentration 40 μg/mL were used.

#### Linearity

The linearity was analyzed through the standard plot of catechin, diosgenin, gallic acid, quercetin, rutin and tannic acid ranging from 0.05–100 μg/mL (Table 1). Different dilutions of standards were prepared by diluting stock solution (1.0 mg/mL) with methanol and analyzed in triplicate. The linearity was evaluated by linear regression analysis, which was calculated by the least-square regression analysis.

**Table 1.** Validation data of developed method for catechin, diosgenin, gallic acid, quercetin, rutin and tannic acid.

S. No.	Metabolites	Linearity range (μg/mL)	Regression equation	Coefficient ofcorrelation	LOD (μg/mL)	LOQ (μg/mL)
1	Catechin	0.05–100	Y = 30539.0X + 3962.7	0.98	0.08	0.24
2	Diosgenin	0.1–100	Y = 2857.9X + 2213.6	0.98	0.17	0.51
3	Gallic acid	1–100	Y = 330.8X − 2222.8	0.98	1.05	3.2
4	Quercetin	0.1–100	Y = 2825.6X + 5561.1	0.99	0.38	1.15
5	Rutin	1–100	Y = 1744.6X + 806.1	0.99	0.91	2.78
6	Tannic acid	1–100	Y = 1816.4X − 953.8	0.99	0.21	0.63

#### Accuracy

In order to check the accuracy of the developed method, solutions of each standard with different concentrations (Table 2) was prepared and analyzed by developed HPLC method. The analysis was carried out in metabolite-plasma solutions, each solution was analyzed in triplicates and the percent recoveries (mean ± SEM with %CV of three replicates) of selected standards (diosgenin, catechin, rutin, gallic acid, tannic acid and quercetin) in drug-plasma solution were calculated.

**Table 2.** Precision and recovery data of developed method for catechin, diosgenin, gallic acid, quercetin, rutin and tannic acid.

Metabolite	Concentration(μg/mL)	Inter-day		Intra-day		Percentage of recovery	
		Mean peak area ± SEM	%CV	Mean peak area ± SEM	%CV	Mean % of recovery ± SEM	%CV
Catechin	1	175466.3 ± 211	0.2	175499.7 ± 0.19	0.19	98.33 ± 0.33	0.58
	40	985346.0 ± 235.2	0.04	985446.0 ± 253.5	0.04	98.64 ± 0.32	0.56
	80	2516146.0 ± 285.7	0.01	2516146.0 ± 230.1	0.01	97.32 ± 0.33	0.60
Diosgenin	1	14381.3 ± 100.6	1.20	14481.3 ± 80.2	0.95	98.16 ± 0.16	0.29
	40	139673.0 ± 216.3	0.26	139773.0 ± 88.1	0.10	98.56 ± 0.08	0.15
	80	249698.0 ± 60.9	0.04	249786.3 ± 51.1	0.03	98.31 ± 0.07	0.13
Gallic acid	10	2599.0 ± 42.5	2.83	2551.0 ± 27.1	1.83	98.50 ± 0.05	0.10
	40	9883.0 ± 23.8	0.41	9937.6 ± 33.4	0.58	98.23 ± 0.08	0.15
	80	23626.3 ± 113.7	0.83	23953.0 ± 41.1	0.29	98.28 ± 0.16	0.28
Quercetin	1	14267.3 ± 44.3	0.53	14300.6 ± 77.6	0.94	98.83 ± 0.16	0.29
	40	112741.3 ± 41.8	0.06	112708.0 ± 35.6	0.05	98.59 ± 0.05	0.08
	80	228678.3 ± 52.3	0.03	228745.0 ± 30.0	0.02	98.40 ± 0.11	0.20
Rutin	10	20524.6 ± 99.1	0.83	20534.6 ± 95.9	0.80	97.40 ± 0.66	1.10
	40	63989.0 ± 72.02	0.19	63965.6 ± 94.2	0.20	97.04 ± 0.48	0.80
	80	146348.0 ± 87.8	0.10	146348.0 ± 66.5	0.10	98.41 ± 0.05	0.09
Tannic acid	10	20603.3 ± 54.4	0.45	20480.3 ± 117.0	0.98	98.80 ± 0.21	0.36
	40	64877.3 ± 50.8	0.13	64947.3 ± 81.02	0.21	98.65 ± 0.15	0.26
	80	146273.7 ± 49.9	0.05	146307.0 ± 0.07	0.14	98.92 ± 0.17	0.31

Data are expressed as mean ± SEM, (*n* = 6).

#### Precision

The precision of the method was determined by repeatability (intra-day precision) and intermediate precision (inter-day precision) of each standard solution separately. Precision was determined in six replicates of the mixed standard solution on the same day (intra-day precision) and daily for six times over a period of one week (inter-day precision). The results were expressed as %CV of the measurements.

#### Sensitivity

Limit of detection (LOD) and limit of quantitation (LOQ) was determined using calibration curve method according to the ICH recommendations (ICH Q2B, 1996). The LOD (*k* = 3.3) and LOQ (*k* = 10) of the proposed method were calculated using the following equation:

A = kσ/S

where *A* is LOD or LOQ, *σ* is the standard deviation of the response and *S* is the slope of the calibration curve.

#### Robustness

To determine the robustness of the developed method, the effect of flow rate was studied at 0.9 and 1.1 mL/min instead of 1.0 mL/min. The effect of column temperature was studied at 25 °C and 35 °C instead of 30 °C. The effect of the initial mobile phase composition of gradient elution was assessed at (ACN: water =5:95, v/v) and (ACN: water =15:85, v/v) instead of (ACN: Water =10:90, v/v). The %CV of robustness testing under these conditions was calculated in all cases.

### Quantitative estimation of diosgenin, catechin, rutin, gallic acid, tannic acid and quercetin in plant extract/fractions

One hundred milligrams of each extract was dissolved in 5.0 mL of HPLC grade methanol separately, transferred into 10 mL volumetric flasks, vortexed for 2 min, ultrasonicated for 5 min and finally volume was filled up to the mark with HPLC grade methanol. Prepared sample solutions were filtered through a 0.45 μM PTFE membrane filter and 10 μL of filtered solutions was injected into HPLC apparatus. Filtered samples were stored at −20 °C until analysis. Separation and analysis of samples diosgenin, catechin, rutin, gallic acid, tannic acid and quercetin were carried out through the newly developed and validated HPLC method. Two different concentrations of each extract (5.0 and 10 mg/mL) were used for HPLC analysis and each sample was analyzed in triplicate and the mean concentration of each metabolite found were calculated from linearity plot.

### Statistical analysis

Values were expressed as mean ± standard deviation (SD). Two-way analysis of variance (ANOVA) followed by ‘Bonferroni posttests’ (Graph Pad, San Diego, CA) was used for statistical analysis. All the treatment groups were compared with the toxic control group. *p* Values <0.05 were considered as statistically significant.

## Results

### Yield of mother extract and fraction

**Figure 1.**
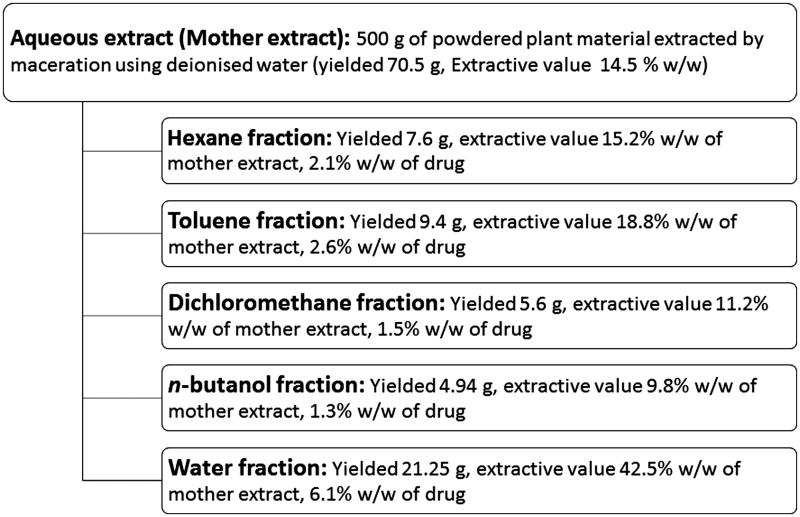
Schematic representation of extraction and fractionation of aqueous extract (mother extract) of gokhru showing extractive values.

The plant material was extracted using deionized water by maceration. The maceration extraction was selected for study due to its high yields and called as mother extract (14.5% w/w).This was further fractionated using hexane (2.1% w/w), toluene (2.6% w/w), DCM (1.5% w/w), *n*-butanol (1.3% w/w) and water (6.1% w/w). However, 5.2 g of mother extract (3.9% w/w) of the drug was lost during the processing (Figure 1). The content of catechin, diosgenin, gallic acid, quercetin, rutin and tannic acid are shown in Table 3.

**Table 3.** Percentage yield of extract and metabolite content in different extract of gokhru.

Extract/Fractions	Yield (%w/w)	Percentage in dried extract (%w/w)					
		Catechin	Diosgenin	Gallic acid	Rutin	Tannic acid	Quercetin
Mother extract	14.5 ± 0.25	3.52 ± 0.54	4.34 ± 0.55	0.97 ± 0.25	3.42 ± 0.24	5.85 ± 0.15	0.99 ± 0.24
Hexane fraction	2.1 ± 0.31^a^	0.004 ± 0.014^a^	0.12 ± 0.23^a^	–^a^	2.8 ± 0.32^c^	0.3 ± 0.45^a^	–^a^
Toluene fraction	2.6 ± 0.78^a^	0.72 ± 0.26^a^	1.29 ± 0.47^a^	0.18 ± 0.14^b^	6.1 ± 0.51^a^	3.46 ± 0.32^a^	–^a^
DCM fraction	1.5 ± 0.17^a^	2.02 ± 0.94^a^	1.23 ± 0.74^a^	0.57 ± 0.47^ns^	10.34 ± 0.44^a^	7.18 ± 017^a^	0.17 ± 0.32^b^
*n*-Butanol fraction	1.3 ± 1.23^a^	2.19 ± 0.47^a^	12.75 ± 0.18^a^	0.77 ± 0.33^ns^	6.12 ± 0.36^a^	15.81 ± 0.47^a^	1.95 ± 0.41^a^
Water	6.1 ± 0.66^a^	5.34 ± 0.65^a^	1.35 ± 0.61^a^	2.03 ± 0.47^a^	5.76 ± 0.27^a^	11.17 ± 0.19^a^	0.78 ± 0.19^ns^

Data are expressed as mean ± SEM, (*n* = 6).

a *p* < 0.001.

b *p* < 0.01.

ns ^c^ *p* > 0.05 as compared to mother extract.

### Antiurolithiatic potential of extract in synthetic urine

**Figure 2.**
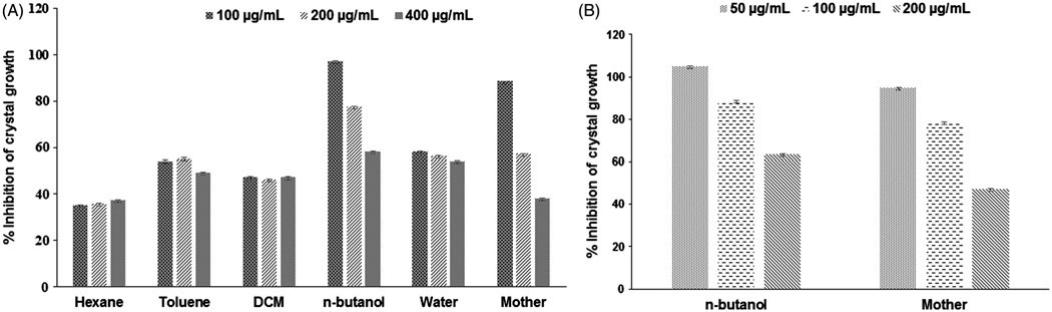
Effect of different concentrations of extract and fractions of gokhru on calcium oxalate crystallization (A) in synthetic urine and (B) in plasma.

Calcium oxalate and calcium phosphate are the two major type of crystals found in kidney stones. Over the period of time, crystals combine to form a small, hard mass called as stones. Further, calcium oxalate stones have classified into two types i.e., calcium oxalate monohydrate stones (COM) and calcium oxalate dihydrate (COD) (Saha & Verma 2013). *n*-Butanol fraction along with mother extract of gokhru has greater capability to dissolve calcium oxalate as a foremost element for stone forming in the urinary tract. In *in vitro* conditions, a lower percentage of mother extract and *n*-butanol fraction indicates more potency in the dissolution of calcium oxalate crystals whereas a higher percentage of toluene fraction indicating more potency in the dissolution of crystals as shown in Figure 2, i.e., the graph of percentage inhibition of the crystallization of calcium oxalate with different concentrations of the mother extract and all the fractions of gokhru.

### Effect of extracts and fractions on nucleation assay

**Figure 3.**
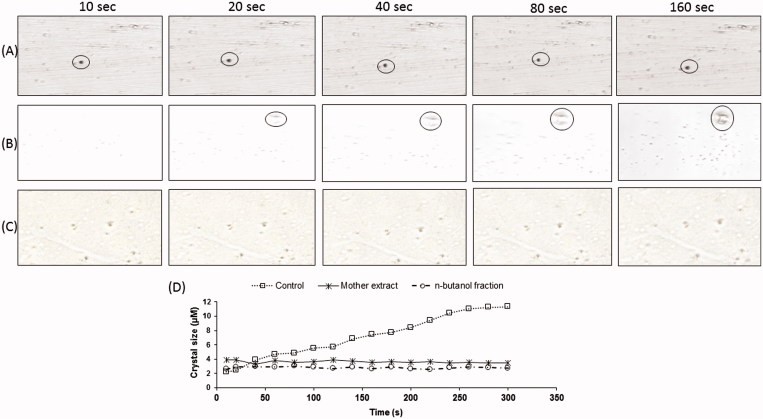
SCIM image sequences of calcium oxalate precipitate showing no increase in crystal size in (A) mother extract (C) *n*-butanol fraction treated and (B) without drug treated reaction solution, (D) calcium oxalate growth curves exhibit the step-like crystals size with respect to incubation time

The first calcium oxalate crystal was observed after 5 min of incubation and then throughout the reaction time the observed crystals were found to be precipitating. For analysis of precipitating crystals, measurements started when a microscopic field having a calcium oxalate crystal was encountered. Crystals, as small as 3.0 μm, could be detected under the microscope due to the high resolution of the imaging method. Figure 3 shows an image sequence of growing crystals under control (Figure 3(B)) and drug-supplemented (Figure 3(A,C)) conditions. In the control condition, the size of the crystals was gradually increased (3.12–11.2 μm) whereas, in drug supplemented reaction, no increase in the size of crystals was observed. Initially, whatever calcium oxalate crystals were formed and further no increment in size of stone was observed in the presence of extract/fractions, concluding that both extract and fractions have preventive action on stone formation rather curative properties to break down the calcium oxalate crystal. Figure 3(D) precisely describes the growth curves of calcium oxalte crystals with relation to their size. The step-like growth curves found for crystals grown under control conditions and it could not also be observed in the presence of either mother extract or *n*-butanol fractions but the growth behavior was remained in steady state. Thus, the study of nucleation assay under confocal microscope have a great scope to study the mechanism of action of drugs having antiurolithiatic potential.

### *Ex vivo* anti-urolithiasis potential of plant extracts

In order to provide the biological environment, the anti-urolithiatic activity of an extract of gokhru was carried out in rat serum. Active fractions along with mother extract were examined by *ex vivo* assay. It was found that the mother extract and *n*-butanol fraction are responsible for the inhibition of calcium oxalate crystals formation. Mother extract showed 88.45% inhibition after 30 min of incubation, whereas *n*-butanol fraction showed 97.25% inhibition. In *ex vivo* conditions, better results were observed than *in vitro*. This phenomenon may be described as inhibition of calcium oxalate crystals, which is due to glycolate oxidase (GOX) as a result of the presence of quercetin (Shirfule et al. 2011). In our analysis, a significant amount of quercetin was found in mother extract and DCM fraction. This *ex vivo* protocol can be used for analysis of the anti-urolithiatic potential of gokhru extract in other biological samples (urine) also.

### Validation of HPLC method

Several mobile phases were tried using various proportions of different aqueous phases and organic modifiers. The best chromatogram was obtained using acetonitrile and 0.5% v/v formic acid in water as a solvent system, using gradient elution mode (Initially 100% A, 0–5 min: 90% A, 5–10 min: 70% A, 10–15 min: 30% A, 15–20 min: 2% A, 20–22 min: 100% A). Different formic acid strengths were tried (0.25 and 1% v/v). Formic acid was substituted by phosphoric acid in some trials. In all these trials, the chromatograms showed broad asymmetric peaks and/or increased retention times and, consequently, fewer theoretical plates for the eluted peaks. Decreased in the initial concentration of formic acid in water (from 100 to 75%), resulted in splitting of the tannic acid chromatogram and overlapping of rutin and catechin chromatogram, though retention time was slightly decreased. Slight increase in the rate of change of acetonitrile concentration (0–5: min 60% A), resulted in no splitting of any chromatogram but chromatogram of rutin and tannic acid was overlapped with increased retention times with excessive peak tailing. Detection and scanning were carried out at their optimum lambda max. Ultimately, a mobile phase consisting of acetonitrile and formic acid (0.5% v/v) was selected for analysis of targeted flavonoids and phenolics content.

#### Linearity

Linearity responses of analyzed metabolites were assessed in the concentration range 0.2–20 μg/mL. The linear equations for the calibration plots and correlation coefficient (*r* ^2^) are shown in Table 1. The range was established with five replicate readings of each concentration.

#### Accuracy

To the earlier analyzed sample, a known concentration of the mixed standard solution of markers (catechin, diosgenin, gallic acid, quercetin, rutin and tannic acid) was used at three different concentrations levels. These solutions were re-analyzed by the developed method and results are shown in Table 2. The recovery of each standard from the prepared samples ranges from 97–99% i.e., within ±1% range. Furthermore, the coefficient of variation (CV) also lies within acceptance range i.e., ≤2.0%. All these observations indicate the accuracy of the developed method, which is because of the narrowness of theoretical and actual yields.

#### Precision

The precision of the method was determined in terms of intra-day and inter-day variations (%CV). Intra-day precision (%CV) was assessed by analyzing the mixed standard drug solutions within the calibration range, three times on the same day, whereas inter-day precision (%CV) was assessed by analyzing the drug solutions within the calibration range on three different days over a period of a week. The results are shown in Table 2.

#### Repeatability

Repeatability of sample application was assessed by multiple injections of 500 ng/mL homogenous sample of standard metabolites that indicate the performance of the HPLC instrument under chromatographic conditions. The %CV of catechin, diosgenin, gallic acid, quercetin, rutin and tannic acid were found to be 0.89, 0.58, 0.78, 0.97 and 0.31%, respectively.

#### Robustness

The robustness is associated with the change in results obtained with the slight variation in the optimum values of the parameters of the developed method. It measures the consistency of the developed method. Here, the robustness of the methods was checked by varying the ratio of the mobile phase, its flow rate and column temperature. Results were measured in terms of per cent change in detector response due to change in method parameters. The results are shown in Table 4. Results indicate that by the deviation of nearly <10% from the optimized initial mobile phase ratio and its flow rate, the %CV was not more than 2.66% for mobile phase flow rate and not more than 2.24% for mobile phase ratio. But, the increase in flow rate and column temperature reduced the retention times proportionately.

**Table 4.** Robustness of the developed method for catechin, diosgenin, gallic acid, quercetin, rutin and tannic acid.

Parameters		Amount metabolite spiked to plasma (μg/mL)	Mean % of recovery ± SEM					
			Catechin	Diosgenin	Gallic acid	Quercetin	Rutin	Tannic acid
Change in initial mobile phase composition (v/v) Initially, acetonitrile: 0.5% formic acid in water (100:0)	(5:95)	40	98.1 ± 0.31	96.5 ± 0.52	97.2 ± 0.31	95.5 ± 0.42	97.5 ± 0.25	96.8 ± 0.24
	(10:90)	40	99.3 ± 0.42	98.8 ± 0.34	99.2 ± 0.44	98.2 ± 0.44	98.4 ± 0.43	98.5 ± 0.21
	(15:85)	40	97.8 ± 0.39	97.2 ± 0.28	96.9 ± 0.28	96.5 ± 0.38	97.5 ± 0.62	96.8 ± 0.35
Change in column temperature (°C)	25	40	97.2 ± 0.24	97.8 ± 0.33	96.5 ± 0.26	96.8 ± 0.43	97.5 ± 0.41	96.5 ± 0.36
	30	40	98.8 ± 0.36	99.2 ± 0.66	98.7 ± 0.45	98.9 ± 0.65	98.5 ± 0.22	99.5 ± 0.45
	35	40	97.7 ± 0.62	96.9 ± 0.53	97.5 ± 0.28	97.5 ± 0.35	96.7 ± 0.35	97.8 ± 0.31
Change in flow rate (mL/min)	0.9	40	97.1 ± 0.53	96.4 ± 0.49	98.2 ± 0.45	97.5 ± 0.41	97.8 ± 0.17	97.5 ± 0.24
	1.0	40	98.4 ± 0.23	98.8 ± 0.51	99.2 ± 0.41	99.7 ± 0.15	98.4 ± 0.15	98.9 ± 0.17
	1.1	40	96.2 ± 0.41	97.2 ± 0.51	97.9 ± 0.44	98.2 ± 0.21	97.5 ± 0.24	96.8 ± 0.18

Data are expressed as mean ± SEM, (*n* = 6).

#### Sensitivity

The LOD and LOQ of the proposed method for selected metabolites are listed in Table 1. The low values of LOD and LOQ indicates the sensitivity of the developed method.

#### System suitability test

**Figure 4.**
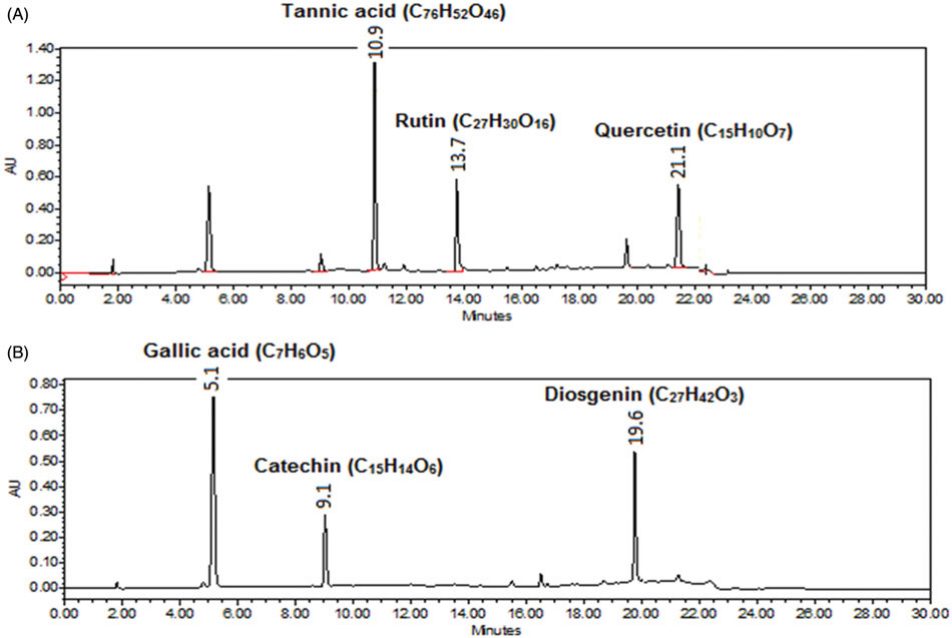
HPLC chromatogram of standard marker compounds at (A) 254 nm (tannic acid, rutin and quercetin) and at (B) 278 nm for gallic acid, catechin and diosgenin.

A solution of standards with a constant volume was injected six times under the developed conditions. The chromatogram at 254 nm (Figure 4(a)) showed that the retention times of quercetin, rutin and tannic acid were 16.2, 12.2 and 9.4 min, respectively. The retention times of catechin, diosgenin and gallic acid were 9.08, 19.6 and 5.09 min, respectively, when chromatogram was recorded at 278 nm (Figure 4(b)). Both the chromatograms showed a better resolution (selectivity >1.9) for all metabolites. The retention times (*R*_t_), theoretical plates (*N*), tailing factor (*T*) and capacity factor are reported in Table 5. The total run time was 25 min.

**Table 5.** System suitability of developed HPLC method for catechin, diosgenin, gallic acid, quercetin, rutin and tannic acid.

System suitability parameters	Proposed method					
	Catechin	Diosgenin	Gallic Acid	Quercetin	Rutin	Tannic acid
Retention time	9.1	19.6	5.1	21.4	13.6	11.0
Capacity factor	0.38	0.67	0.87	0.14	0.65	0.18
Theoretical plate	4338	2458	2987	8794	3547	4254
HETP (mm)	0.021	0.034	0.047	0.019	0.039	0.024
Peak asymmetry @ 10% height	1.15	1.56	1.32	2.15	1.89	1.12
Tailing factor	1.12	1.20	1.21	0.87	1.87	0.98

### Sample preparation for HPLC analysis of diosgenin, catechin, rutin, gallic acid, tannic acid and quercetin in blood

**Figure 5.**
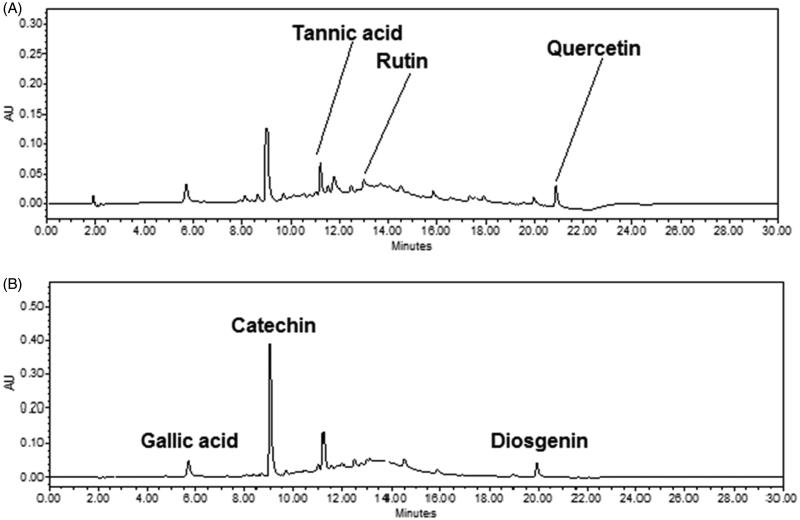
HPLC chromatogram of extract at (A) 254 and (B) 278 nm indicating the presence of marker metabolites (tannic acid, rutin and quercetin, gallic acid, catechin and diosgenin) in mother extract of gokhru.

The addition of organic solvent with or without acid or base to serum sample caused precipitation of protein due to denaturation, and metabolites were dissolved in the liquid phase (Kole et al. 2011). Protein precipitation from serum sample with methanol resulted in 89% recovery of all metabolites. But by using acidified methanol, we achieved more than 91% recovery of flavonoidal metabolites (rutin) (Zhang et al. 2009) and basified methanol, showed higher extraction efficiency for alkaloidal metabolites (Patil et al. 2012). But, both acidification and further basification of serum samples increased extraction recovery (93–98%) of all targeted metabolites. In liquid–liquid extraction, maximum recovery (95%) of all the metabolites was achieved by using a mixture of ethyl acetate, methyl-tert-butyl-ether and acetonitrile in a ratio of 1:1:1, v/v/v. A satisfactory recovery was obtained using methanol but purity was not satisfactory. By using only ethyl acetate, methyl-*tert*-butyl-ether and mixture of ethyl acetate and isopropyl alcohol, less than 70% recovery was obtained and these organic solvents were preferred in previous research work for liquid–liquid extraction of flavonoidal, phenolic and alkaloidal metabolites (Liu et al. 2011; Xu et al. 2013; Zhang et al. 2013). The selection of the organic solvent for liquid–liquid extraction of metabolites was based on recovery and purity of organic layer (Yun et al. 2013). Final sample preparation followed pre-conditioning of serum sample (Figure 5) and extraction through mixed solvents composed of methyl-tert-butyl-ether, acetonitrile and ethyl acetate with a ratio of 1:1:1, v/v/v, which resulted in more than 95% recovery of all the targeted metabolites. Recovery of metabolites through liquid–liquid extraction is shown in Table 6.

**Table 6.** Liquid–liquid extraction of catechin, diosgenin, gallic acid, quercetin, rutin and tannic acid from serum.

Solvent used	Percentage recovery					
	Catechin	Diosgenin	Gallic acid	Quercetin	Rutin	Tannic acid
Solvent selection						
Ethyl acetate	87.3 ± 0.12^a^	91.2 ± 0.16^b^	25.2 ± 0.16^a^	85.3 ± 0.33^a^	84.3 ± 0.31^b^	41.2 ± 0.15^a^
Methyl-*tert*-butyl ether	61.2 ± 0.21^a^	82.3 ± 0.14^a^	52.3 ± 0.25^a^	48.2 ± 0.16^a^	87.6 ± 0.33^b^	58.3 ± 0.16^a^
Ethyl acetate: isopropyl alcohol (1:1, v/v)	32.9 ± 0.14^a^	61.7 ± 0.41^a^	71.5 ± 0.31^b^	47.3 ± 0.11^a^	41.1 ± 0.41^a^	34.2 ± 0.33^a^
Acetonitril:methyl-*tert*-butyl-ether:ethyl acetate (1:1:1, v/v/v)	92.2 ± 0.34^a^	95.4 ± 0.21^b^	93.7 ± 0.25^a^	94.6 ± 0.26^a^	93.4 ± 0.42^a^	96.5 ± 0.16^a^
Methanol	85.6 ± 0.24^b^	78.5 ± 0.32^a^	72.9 ± 0.23^a^	84.3 ± 0.24*	73.5 ± 0.33^a^	68.2 ± 0.12^a^
Preconditioning of solvent						
Basification	37.3 ± 0.33^a^	70.5 ± 0.33^a^	48.2 ± 0.16^a^	87.3 ± 0.32^b^	82.7 ± 0.18^a^	87.6 ± 0.25^b^
Acidification	91.2 ± 0.17^a^	38.4 ± 0.24^a^	84.2 ± 0.13^b^	21.5 ± 0.21^a^	41.6 ± 0.17^a^	41.2 ± 0.13^a^
Basification and acidification	98.4 ± 0.32^a^	96.2 ± 0.31^a^	95.5 ± 0.17^a^	96.5 ± 0.19^a^	95.5 ± 0.25^a^	95.6 ± 0.27^a^

Data are expressed as mean ± SEM, (*n* = 6).

a *p* < 0.001.

b *p* < 0.0.

## Discussion

There is growing evidence that calcium oxalate nephrolithiasis is concomitant with renal injury. Hyperoxaluria is a key risk factor for calcium oxalate nephrolithiasis, and calcium oxalate is the most common type of urinary stone. High amount of oxalate in kidney caused a number of changes in the renal epithelial cells, such as an increase in free radical production and a decrease in antioxidant status, followed by cell injury and cell death. These changes are the important predisposing factors for the acceleration of crystal adherence and retention (Moriyama et al. 2007).

Due to several side effects and failure to prevent recurrence by the present day treatment therapy for urolithiasis, alternative treatment through herbal medicine have assumed a great importance. A histrionic advancement in using phytotherapy for urolithiasis treatments has been observed in recent years and many researchers from various fields have proposed to further scientific study on its efficacy. Many medicinal plants have been used for centuries for the treatment of urinary stones in spite of having any rationale behind their use. In view of its medicinal use, gokhru fruits aqueous extract and its fractions were studied to evaluate its anti-urolithiatic potential using different models. The inhibitory potency of the plant was tested on the nucleation and growth of the most commonly occurring kidney stones, calcium oxalate monohydrate. A concentration-dependent trend of inhibition was observed using mother extract of gokhru fruits and its *n*-butanol fraction with maximum inhibition of 88.5% and 97.3%, respectively, for calcium oxalate growth assay. Our reports are in agreement with the studies previously reported on anti-urolithiatic potency of gokhru on the growth of COM crystals using double diffusion gel growth technique (Joshi et al. 2005).

To provide biological environments, *ex vivo* anti-urolithiatic activity was carried out in blood plasma. This *ex vivo* study represents one of the first use of plasma rather than using synthetic urine to check anti-urolithiatic potential of any herbal formulation. In the present *ex vivo* finding, significantly better results have been observed as compared to synthetic urine. This *ex vivo* study can be used as a model to check anti-urolithiatic potential of extracts/medicine. Some drugs may give results *in vitro* but it may not work in *in vivo* model. While performing *ex vivo* activity, the results will facilitate to *in vivo* also. Before doing *in vivo* activity, one can check its effect on *ex vivo* model also. This study may work as a model to elaborate its mechanism behind its activity under controlled environment.

Confocal microscopy has been used to study the crystallization kinetics of calcium oxalate monohydrate (COM) and calcium oxalate dehydrate (COD). Whereas control samples generated well facetted monoclinic COM and increase in the proportion of COD crystallized and increase particle number density. The inhibition of COM growth by extract/fractions may be characterized by decreased displacement velocities. Analysis of image sequences suggested that crystal growth is strongly affected by extract/fractions. This direct visualization helps to reveal the anti-urolithiatic potential of gokhru by conforming both the *n*-butanol and mother extracts, which can be used for preventive purpose rather than curative properties. The present study represents one of the first uses of confocal microscope for the investigation of fast crystallization processes and growth of calcium oxalate crystals (Grohe et al. 2006). Our findings provide proof of the concept by confirming previous studies performed using scanning inference confocal microscope; in addition, real-time data was presented, in particular, from individual crystals that could not be obtained by traditional *in vitro* methods. Thus, it can be observed, controlled, interpreted and predicted the effect of any medicine on growth/inhibition of calcium oxalate/any other targeted crystals through a confocal microscope. The plant being of edible nature with a long history of medicinal use in the traditional system is reflected to be relatively safe, however, in-depth studies on its safety profile are needed before recommending for clinical use.

**Figure 6.**
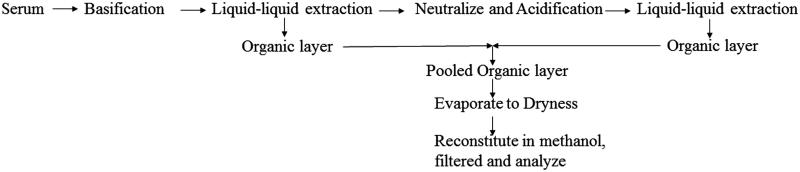
Optimized extraction method for extraction of metabolite marker from serum.

Diosgenin, catechin, rutin, gallic acid, tannic acid and quercetin are the common metabolites found in fruits of gokhru. Qualitative and quantitative analysis of such compounds are getting more important for the evaluation of quality control as well as to define other pharmacological properties such as pharmacokinetics, metabolic pattern recognition. Though, there are several HPLC fingerprinting analysis of this plant extracts reported in the literature, but these are not marker specific (Kumar 2012; Soni et al. 2014). Although same plant from different varieties may have similar categories of metabolites, they might have varied in their content. For that reason nowadays, the fingerprinting analysis is not sufficient for the evaluation of quality control of any specific metabolite and, therefore, there is an urgent need for a method of analysis, which can define a plant in the more specific way and can be used for other purpose also. In the present study, a simple, low-cost and sensitive RP-HPLC method was developed and validated to quantify diosgenin, catechin, rutin, gallic acid, tannic acid and quercetin in extract and fractions of gokhru. During the method development, mobile phase components and ratios, flow rate, column temperature, solvent additives and wavelength were checked to find out the optimum conditions for the best separation of said metabolites. To present the gradient system, different combinations of water with 0.5% v/v formic acid (A) and acetonitrile (B) were attempted. The gradient system in the ratio of A:B, 100:0 at 0 min, 90:10 at 5 min, 70:30 at 10 min, 30:70 at 15 min, 2:98 at 20 min and 100:0 at 22 min, achieved the best separation and was chosen as the gradient solvent system. Both formic acid and acetic acid with 0.5% v/v were tried and best resolute peaks separation achieved with 0.5% v/v formic acid. The initial concentration of A affected the separation of rutin, catechin and tannic acid. The sensitivity of detection of metabolites varies with the changes of wavelength and which was selected by evaluating peak area at optimum conditions. Maximum absorptions were observed at 254 nm (for rutin and quercetin), 278 nm (for diosgenin, gallic acid, tannic acid and catechin). To eliminate the deviation with regards to temperature differentiation, column temperature was controlled and set to 30 °C. The flow rate was arranged to 1.0 mL/min after trials of different flows. The developed and validated RP-HPLC method was used for the analysis of selected metabolites in plant extract/fractions. Further, it was explored in blood plasma. Figure 6 shows the presence of diosgenin, catechin, rutin, gallic acid, tannic acid and quercetin in mother extract and these metabolites were varied significantly (*p* < 0.01) from fractions to fractions.

## Conclusions

In summary, the bioactive fraction (*n*-butanol fraction) in the mother extract of gokhru has the protective capacity rather than the curative properties against urolithiasis. Simultaneous estimation of six common metabolites resulted in a systematic approach for quality control evaluation and it support for biological activity against urolithiasis in a mechanistic way. This study will help for the further discovery of a novel phytochemical from the *n*-butanol fraction of mother extract, whereas simultaneous estimation will help for a metabolic and pharmacokinetic study. *In vitro* and *ex vivo* studies will be the new approach for the determination of anti-urolithiatic potential, which can be used for other drugs as well.
